# Isothermal Time-Temperature-Precipitation Diagram for an Aluminum Alloy 6005A by *In Situ* DSC Experiments

**DOI:** 10.3390/ma7042631

**Published:** 2014-03-28

**Authors:** Benjamin Milkereit, Lydia Giersberg, Olaf Kessler, Christoph Schick

**Affiliations:** 1 Faculty of Mechanical Engineering and Marine Technology, Chair of Materials Science, University of Rostock, Rostock 18051, Germany; E-Mails: lydia.giersberg@uni-rostock.de (L.G.); olaf.kessler@uni-rostock.de (O.K.); 2 Polymer Physics Group, Institute of Physics, University of Rostock, Rostock 18051, Germany; E-Mail: christoph.schick@uni-rostock.de; 3 Interdisciplinary Faculty, University of Rostock, Rostock 18051, Germany

**Keywords:** differential scanning calorimetry (DSC), aluminum alloy 6005A, isothermal time-temperature-precipitation diagram, TTP diagram

## Abstract

Time-temperature-precipitation (TTP) diagrams deliver important material data, such as temperature and time ranges critical for precipitation during the quenching step of the age hardening procedure. Although the quenching step is continuous, isothermal TTP diagrams are often applied. Together with a so-called Quench Factor Analysis, they can be used to describe very different cooling paths. Typically, these diagrams are constructed based on mechanical properties or microstructures after an interrupted quenching, *i.e*., *ex situ* analyses. In recent years, an *in situ* calorimetric method to record continuous cooling precipitation diagrams of aluminum alloys has been developed to the application level by our group. This method has now been transferred to isothermal experiments, in which the whole heat treatment cycle was performed in a differential scanning calorimeter. The Al-Mg-Si-wrought alloy 6005A was investigated. Solution annealing at 540 °C and overcritical quenching to several temperatures between 450 °C and 250 °C were followed by isothermal soaking. Based on the heat flow curves during isothermal soaking, TTP diagrams were determined. An appropriate evaluation method has been developed. It was found that three different precipitation reactions in characteristic temperature intervals exist. Some of the low temperature reactions are not accessible in continuous cooling experiments and require isothermal studies.

## 1. Introduction

The most important heat treatment to increase the strength of aluminum alloys is precipitation hardening, which is composed of three steps: solution annealing, quenching and ageing. The maximal strength values can be achieved only when age hardening aluminum alloys are quenched above a critical rate. If quenching is too slow, coarse precipitates will form during cooling. Those coarse precipitates do not increase the strength, but reduce the remaining solid solution available for the growth of strengthening precipitates during ageing.

For practical heat treatment, as well as for heat treatment simulation of metals, their quench sensitivity, *i.e*., the transformation (respectively precipitation) behavior depending on the process temperature and duration, must be known. For steels, a broad range of isothermal time-temperature-transformation (TTT) diagrams and continuous TTT diagrams exist [1], because they can be determined by *in situ* dilatometric methods. However, for aluminum alloys, such time-temperature-precipitation (TTP) diagrams have been scarcely available in the past, due to the lack of suitable *in situ* characterization methods. Only a few isothermal TTP diagrams for aluminum alloys exist; these have been determined by *ex situ* characterization methods.

Both types of TTP diagram are related to the quenching step within the age hardening procedure. Continuous cooling precipitation or transformation diagrams provide the required information directly for certain defined cooling paths. From isothermal TTP diagrams, similar information about continuous cooling processes is available for very different cooling paths by applying Quench Factor Analysis [2] based on Scheil’s rule of additivity [3,4]. This is often used by heat treatment simulation software in order to calculate the microstructural evolution during a cooling process. Simir and Gür mention in [5] that “the most straightforward approach for calculating microstructural evolution during a continuous cooling process would simply be to introduce CCT diagrams into the computer program. A CCT diagram is valid only for the exact temperature histories used to draw it, and the cooling curves are normally plotted on the diagram. However, during quenching, the cooling rate at a point is generally not constant and hence, it does not follow one of those curves; therefore, the CCT diagram is no longer valid. […] As a workaround, Scheil’s additivity principle is commonly employed to relate a TTT diagram to the transformation behavior for an arbitrary continuous cooling path […]. Thus, the cooling curve can be treated as a series of small isothermal time steps connected by instantaneous temperature jumps […].” Currently, the isothermal TTP diagrams for aluminum alloys are typically based on the analysis of the microstructure or mechanical (resp. corrosion) properties after quenching several samples to different temperatures and soaking for different periods of time (see, for example, [6–14]). The microstructure approach requires an extensive series of high-resolution microstructure analyses. These diagrams may deliver time-temperature-precipitation diagrams that show different precipitation phases and nucleation sites [15,16]. The possibility that is more frequently used is to measure certain properties, such as yield strength or hardness [10,11,13,14]. This approach requires less effort. However, the related time-temperature-property diagrams typically are not able to resolve different precipitation phases. Rather, they typically show one so-called “C-curve” related to a certain property.

Some publications have shown that it is possible to measure *in situ* precipitation reactions at an isothermal soaking step with different calorimetric techniques [17–23]. However, typically, the whole heat treatment was not performed in the calorimeter device. Solution annealing and quenching were performed outside the calorimeter, for example in a salt bath or in an air circulating furnace. For the isothermal soaking step, the samples were inserted in a preheated calorimeter. Hence, a relatively large time step cannot be measured, because the temperatures of the sample and furnace have to equilibrate first.

This study therefore aims to develop a method that enables the measurement of isothermal TTP diagrams of aluminum alloys by *in situ* differential scanning calorimetry (DSC) measurements. Thus, the whole heat treatment will be performed in the DSC and it will be possible to follow precipitation reactions during the isothermal soaking procedure at selected temperatures.

The precipitation processes that occur are related to relatively weak exothermic reactions. Hence, a highly sensitive measuring system is necessary. During recent years, a highly sensitive *in situ* calorimetric method to record continuous TTP diagrams for aluminum alloys has been developed by our group to the application level [24–34].

In this work, we will transfer the knowledge of the continuous cooling work to isothermal measurements of precipitation reactions in aluminum alloys using DSC. In particular, the aim is to perform the whole heat treatment and measurement cycle in a DSC without changing the device, in order to ensure the highest sensitivity, but also to avoid reductions in the measurable time range. A procedure for a reliable evaluation of isothermal time-temperature-precipitation diagrams will be suggested.

This work is focused on the cooling step of the age hardening procedure by developing a new measurement technique that enables the measurement of isothermal TTP diagrams by *in situ* DSC, rather than by extensive *ex situ* analysis. This may eventually lead to an optimization of the simulation of cooling during heat treatment simulations in the future.

Another issue associated with this calorimetric technique is of more fundamental scientific importance. Zhuravlev *et al.* [35] investigated the crystallization of poly(ε-caprolactone) out of the liquid state and mentioned that the temperature range below the maximum of crystallization rate (which is comparable to the nose in isothermal TTP diagrams for aluminum alloys) is generally not accessible for non-isothermal cooling experiments, because the sample becomes amorphous (or supersaturated in the case of aluminum alloys) at the required cooling rates. Isothermal experiments after fast quenches extend the temperature range down to, and below, the glass transition temperature. This problem is basically also valid for the investigation of precipitation reactions from a supersaturated solid solution. Therefore, isothermal DSC experiments, which directly follow a cooling process that is faster than the critical cooling rate, enable the investigation of precipitation below the commonly known “nose” of a C-curve.

## 2. Materials and Methods

### 2.1. Material Investigated

Material from an industrial extruded profile of EN AW-6005A was investigated. This alloy was chosen, because it possesses a critical cooling rate of about 6.3 K/s [27,31], which can be achieved within the DSC device that was used. Pure aluminum (99.9995% Al) was used as the reference. The chemical composition is given in Table 1. The main alloying elements possess an atomic fraction of approximately 0.63% Mg and 0.65% Si. With respect to the quasi-binary composition of Mg_2_Si, the alloy 6005A therefore has an excess of Si.

### 2.2. Measurement Procedure and Data Evaluation

**Figure 1.**
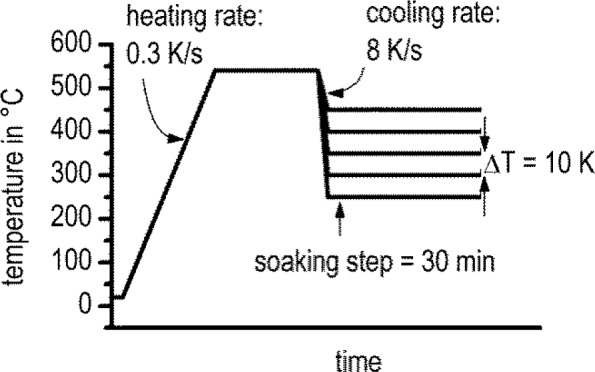
Temperature program of the differential scanning calorimetry (DSC) measurements.

After solution heat treatment at 540 °C for 20 min, the samples were quenched to a certain soaking temperature in a power-compensated DSC (Pyris 1 DSC, PerkinElmer, Waltham, MA, USA). The temperature program and the parameters used are shown in Figure 1. Cooling should be carried out at a rate faster than the critical cooling rate for the alloy [31]. The selected soaking temperatures ranged from 250 °C to 450 °C with temperature intervals ∆*T* = 10 K. The soaking time was determined with preliminary tests, varying the soaking step between 30 and 60 min. The preliminary tests showed that after 30 min, dissolution reactions were no longer detectable. One disc-shaped sample of EN AW-6005A with a diameter of 5.7 mm and a height of 1 mm was repeatedly used for all experiments. Due to initial solution annealing and quenching, a fresh supersaturated solid solution was adjusted for every new experiment. The sample mass was 69.615 mg.

For the investigation of weak precipitation reactions within aluminum alloys, the alloyed sample was scanned in the DSC and compared to an inert reference sample [27]. This reference ideally should have the same absolute heat capacity as the sample, and pure aluminum is commonly used.

**Figure 2.**
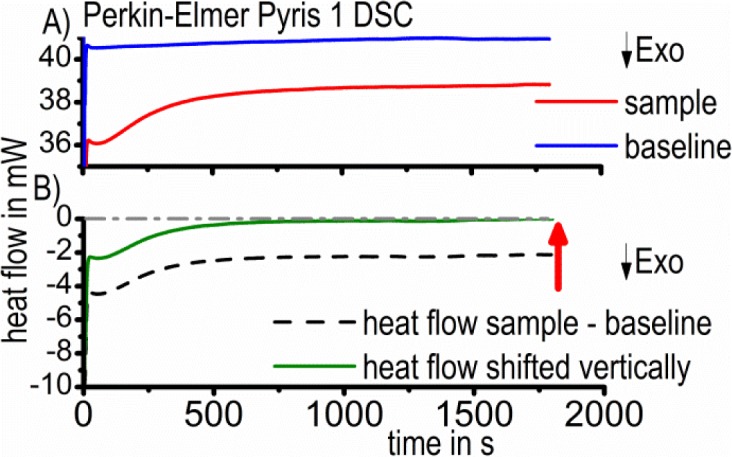
(**A**) The subtraction of the baseline from the sample heat flow curve; (**B**) The vertical shift of the resulting heat flow curve to zero at infinite time.

If the baseline measurement is also performed with two inert reference samples, the value of the excess specific heat capacity results after data evaluation [27]. This evaluation usually includes normalizing the heat flow values by the scanning rate and sample mass to the value of the specific heat capacity. This allows the comparison of different experiments with varying scanning rates. In this work, it was not possible to normalize the heat flow by the scanning rate, as the scan rate of the soaking step equals zero. Therefore, the isothermally measured heat flow after scanning an alloyed sample *versus* a pure aluminum reference sample was used for evaluation. The heat flow curve obtained from the same temperature time profile for two pure aluminum samples was subtracted as a baseline measurement. This baseline subtraction is necessary to distinguish the sample heat flow from instrumental artefacts. Due to small asymmetries between the baseline and sample measurement (e.g., due to different sample or lid positions [36]), this does not necessarily result in a zero value in reaction-free time ranges. However, one basic assumption for the evaluation is that heat flow equals zero as long as no reactions are occurring in the sample. Therefore, the heat flow curves were vertically shifted with respect to time ranges where no reactions are expected (Figure 2).

**Figure 3.**
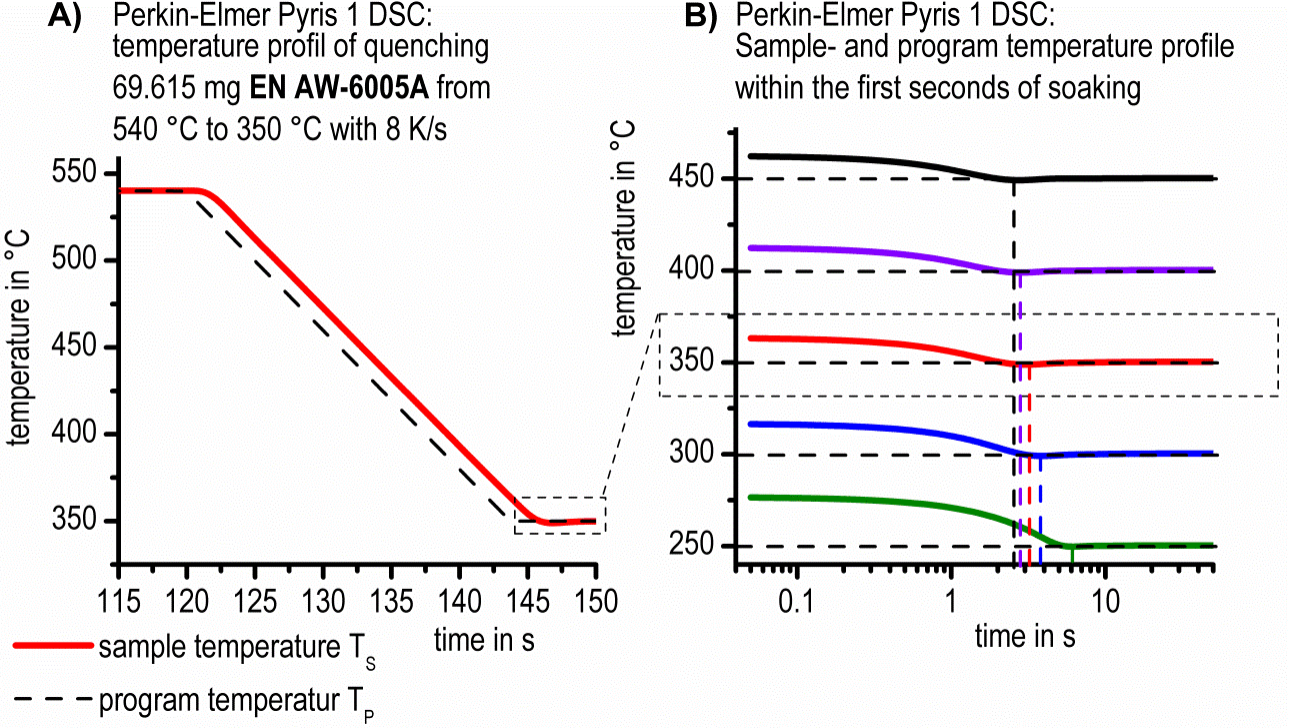
(**A**) Sample and program temperature for a quenching experiment of 69.615 mg EN AW-6005 from 540 °C to isothermal soaking at 350 °C in a Perkin Elmer Pyris 1 DSC; (**B**) Sample and program temperature for the first seconds of isothermal holding at different soaking temperatures. In (**B**), the zero value of the time axis corresponds to the start of isothermal soaking according to the program temperature.

At the beginning of the soaking step, the precipitation reaction may start immediately. Therefore, it may barely be possible to evaluate the precipitation onset directly. Besides, during quenching from the solution treatment to soaking temperature, a temporal delay between the sample temperature and the program temperature occurs. This fact influences the definition of the onset-point of the precipitation reaction. Furthermore, the duration of the quenching step is longer when the soaking temperature is lower. All these effects have been taken into account in the evaluation (Figure 3).

Additionally, a control problem within the DSC measurement at high quenching rates must be considered. The maximum cooling rate possible is dependent on the temperature difference between the DSC furnace and the cold block, which necessarily decreases during the cooling process. Therefore, the scan rate may fall to below the programmed value. When the actual cooling rate of the experiment switches from the cooling step to zero during isothermal soaking, it is inevitable that cooling becomes slower than any critical cooling rate. Hence, a reasonable starting point must be defined.

The continuous cooling precipitation diagram of the batch of aluminum alloy 6005A investigated here is available [31]. Out of this diagram, one can read the critical cooling rates. Two main temperature ranges exist that must be considered: high-temperature (HTR) and low-temperature (LTR) precipitation reactions [27,31]. Each of those has its own known critical cooling rate [27,31]. The critical cooling rates of 6005A are 6.3 K/s for the LTR and 0.67 K/s for the HTR.

**Figure 4.**
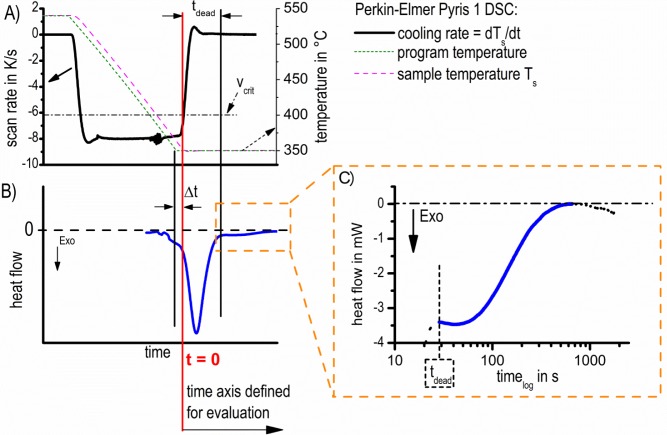
Determination of the intersection point between the critical cooling rate and the actual cooling rate defines *t* = 0. (**A**) The scan rate and temperature; (**B**) Heat flow, the definition of ∆*t* and *t*_dead_ is shown schematically; (**C**) The part of the measured heat flow curve that is suitable for evaluation. The heat flow is plotted on the new defined time axis.

Due to the fact that the cooling rate must fall below these values at the end of the cooling step, precipitation might start already during cooling. Additionally, the change in scanning rate between cooling and soaking leads to a heat flow overshoot, which is an instrumental measurement artefact (the large peak in the blue curve in Figure 4B). This artefact might lead to errors in the interpretation of the DSC curves and, therefore, must be excluded from the evaluation. As the overshoot peak might overlap the reaction onset, a critical dead time (*t*_dead_), which cannot be analyzed, exists. An appropriate way of defining a reliable starting point, which determines the time limits for signal evaluation (cut of overshot artefacts), is schematically shown in Figure 4.

Figure 4 displays a typical temperature and scanning rate profile of the DSC measurements. In Figure 4A the profiles of program- and sample-temperature are plotted corresponding to the right ordinate. Additionally the first derivative of sample-temperature with respect to time, which represents the actual scanning rate, is plotted corresponding to the left ordinate, both as function of time. The alloy-specific critical cooling rate (which further depends on high- or low-temperature-reactions) is denoted by a dash-dot horizontal line.

At the end of the quenching step, the intersection point between the actual applied scanning rate and the critical cooling rate (the red line in Figure 4) was defined as the zero time for the evaluation of isothermal precipitation kinetics in this work. According to this defined zero point, a time interval, ∆*t*, results, according to Figure 4. In order to enable reliable curve evaluation, the isothermal heat flow curves must be shifted horizontally to the newly-defined starting point by ∆*t*.

**Figure 5.**
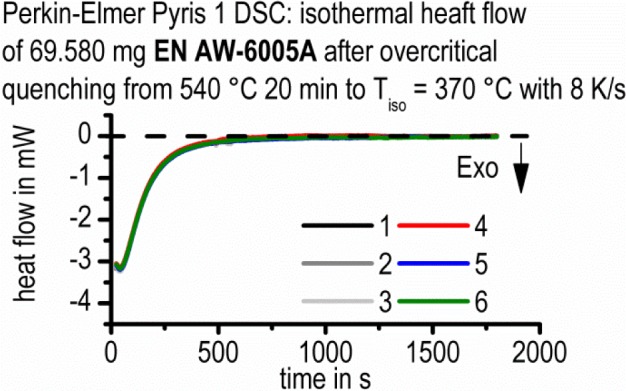
Reproducibility of the measurements indicated by six identical measurements.

In Figure 4B, the corresponding heat flow signal is plotted; however, the accordance between the time axis in Figure 4A and Figure 4 is only schematic (Figure 4A: linear time scale; Figure 4: logarithmic time scale). As a consequence of the transient response of the heat flow, at the moment when the process changes from cooling to soaking, a big heat flow peak artefact results at the beginning of the soaking step: the so-called overshoot peak. This overshoot should not be involved in the evaluation. The critical dead time, *t*_dead_, is defined by the time when the scanning rate is stabilized to zero. The exothermic effect following the overshoot-peak is caused by the precipitation reaction under study. To prove this fact, six measurements with the same parameters were performed. At any of these measurements, nearly identical heat flow curves were observed (Figure 5), showing that the reproducibility of the precipitation reactions in the heat flow curves is appropriate. In some cases, the heat flow drifts for long soaking durations, even though the reaction is expected to be finished. Because of that, these data ranges were excluded too (an example is shown in the right part of the heat flow curve in Figure 4C).

### 2.3. Determination of Isothermal TTP Diagrams

**Figure 6.**
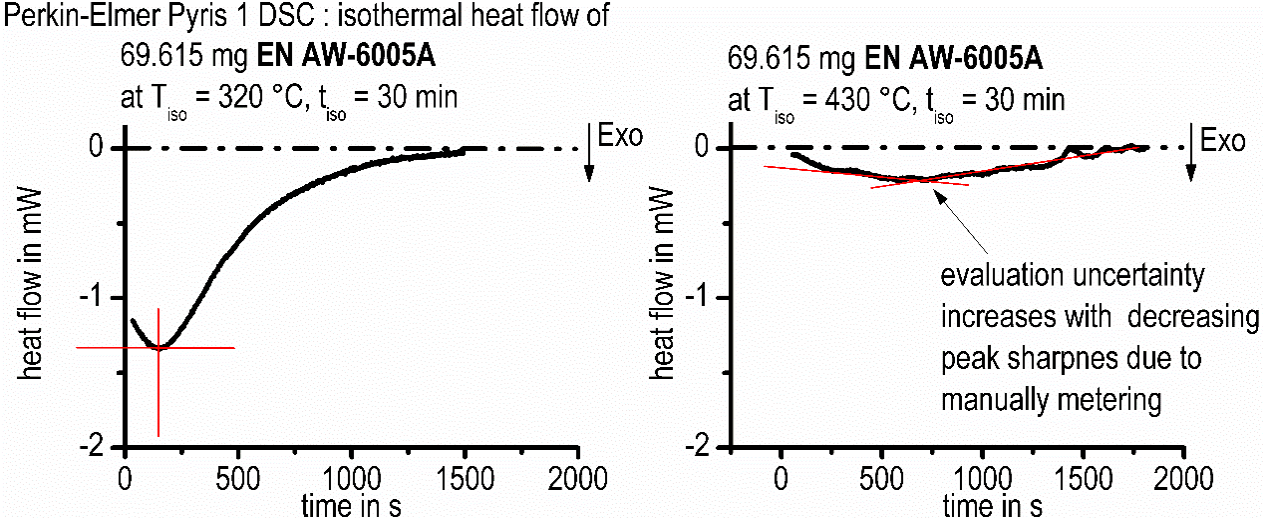
Determination of peak-times.

The aim of this work is to create isothermal TTP diagrams based on *in situ* DSC measurements. Therefore, the characteristic time values of the precipitation reaction, *i.e*., the start and end of the reaction, must be evaluated from heat flow curves like those shown in Figure 4C. However, due to the initial critical dead time mentioned above, as well as the flat course of the heat flow curves at the end of the analyzed time period, a well-adapted further evaluation is necessary. Two options have been developed to characterize the precipitation kinetics for the TTP diagrams: The first is to determine the heat flow peak-time and to plot the corresponding time/temperature values in a time-temperature diagram. If needed, straight lines were fitted to the peak flanks. In those cases the intersection point of the lines was evaluated as the peak-time (Figure 6). This method is relatively easy and fast. However, uncertainties occur with the decreasing peak-sharpness of the heat flow, and only one single time value results for each measured curve.

The second method to create TTP diagrams out of the isothermal DSC measurements is to use a certain relative amount of released heat of any heat flow curve and to plot the corresponding time values of the relative amounts in the time-temperature diagram. Therefore, the total specific precipitation heat or specific enthalpy was calculated by integrating the area below the heat flow curves and normalizing the gained values by the sample mass.

Due to the data evaluation explained above, the evaluable heat flow curves do not start at zero time. Between the defined start point and the onset of the evaluable heat flow curve, a time range exists where the development of the heat flow is unknown. This non-evaluable dead time, *t*_dead_, amounts to an average value of about 30 s (maximum value, 77 s).

**Figure 7.**
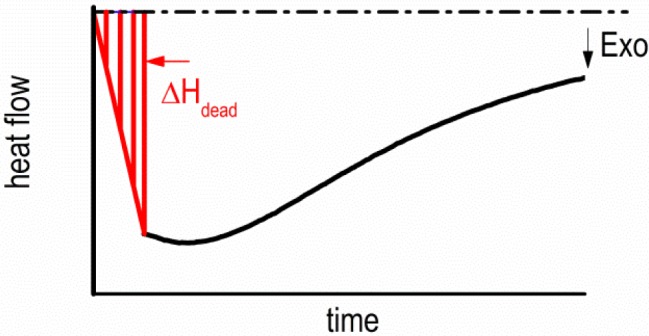
Approximation of specific precipitation heat area ∆*H*_dead_ by a triangle area for the critical dead time, *t*_dead_.

However, the area below this unknown part of the heat flow contributes to the total specific precipitation heat transformed by an unknown value, ∆*H*_dead_. To minimize the error of the total specific precipitation heat, an approximation of the missing ∆*H*_dead_ by a triangle area is suggested (Figure 7). Related to the overall precipitation heat, ∆*H*_dead_ has an average value of 1.6% and a maximal value of 7.5%. For the characterization of the precipitation kinetics in the isothermal time-temperature-precipitation diagram, the values of 10%, 50% and 90% of the observed total specific precipitation heat were chosen.

**Figure 8.**
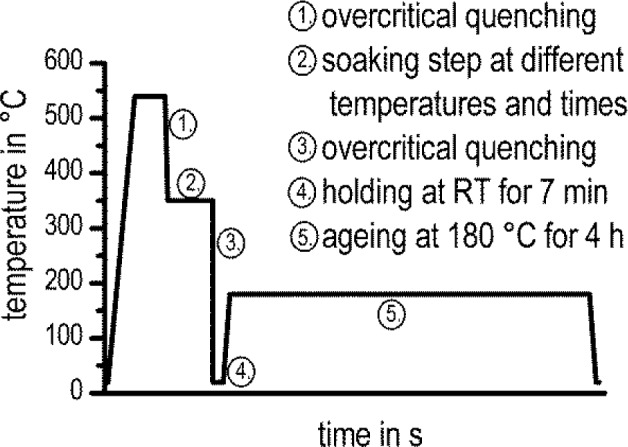
Temperature program for the hardness tests.

In order to understand the way in which the mechanical properties were influenced by the precipitation during the soaking step, the residual hardening potential was analyzed by *ex situ* hardness tests (Vickers hardness HV1). These tests were performed after selected soaking temperatures and times and further overcritical quenching and ageing. Therefore, the samples were aged at 180 °C for 4 h after the DSC-measurements. The temperature program before the hardness test is shown in Figure 8.

## 3. Results and Discussion

**Figure 9.**
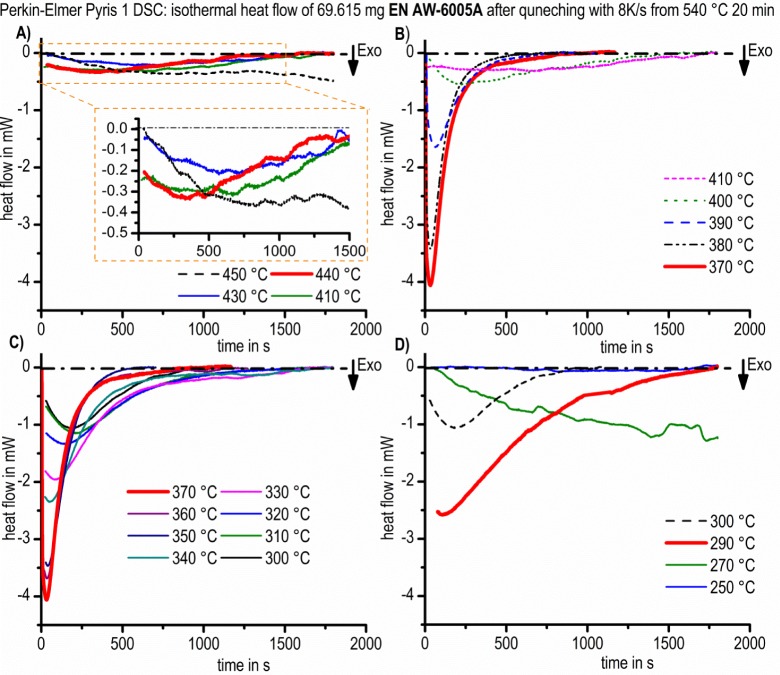
Heat flow curves of alloy 6005A after overcritical quenching from 540 °C for 20 min to temperatures between 450 °C and 250 °C. (**A**) 450–410 °C; (**B**) 410–370°C; (**C**) 370–300 °C; (**D**) 300–250°C.

The recorded and evaluated heat flow curves are shown in Figure 9A–D. In order to ensure readability, the curves are separated into four temperature ranges. All diagrams are scaled in the same way to enable the reader to see the differences in the signal intensity directly, which are dependent on temperature and time. The heat flow shows one peak in each case. This peak shifts in time, and its peak height changes. Three maxima of the heat flow values, within a certain temperature range, were detected. The first temperature area ranges from 450 to 430 °C; the second from 430 °C to 300 °C; and the third from 300 °C to 250 °C. Additionally, the heat flow curves of the second temperature range were separated into two diagrams (Figure 9B,C) following the order of increasing or decreasing intensity, respectively. Within these diagrams, increasing peak values are shown by different solid lines until the maximum peak value is reached. The maximum is highlighted with a solid fat red line. The heat flow curves, where the peak value decreases, are plotted as dashed lines.

For the high temperature range between 450 °C and 430 °C, the maximum peak value is located at 440 °C after about 370 s (Figure 9A). Sluggish reactions with low intensities were observed. Within the high-temperature range, the differences between the heat flows curves are relatively small. The interpretation, therefore, must be carried out with care. To enable the reader to see the differences between the single curves, an additional diagram with different scaling is inserted into Figure 9A.

In the second temperature range from 430 °C to 300 °C, the differences in the heat flow curves are much more pronounced, Figure 9B,C. The maximum heat flow peak was observed at 370 °C after only 33 s. The peak height values and their positions are shifted to longer durations with both higher and lower temperatures. This effect is stronger towards higher soaking temperatures. Within the soaking temperature range from 380 °C to 350 °C, the differences between the heat flow curves are small and the peak height values are the highest (about −0.05 W/g to −0.06 W/g). The reactions are also relatively fast in this temperature range. The peak values were found after around 33 s to 41 s.

In the third temperature range from 300 °C to 250 °C, the precipitation reaction with the highest heat flow peak took place at the soaking temperature of 290 °C and after a period of about 105 s (Figure 9D). In the temperature range of 300 °C to 250 °C, all other peak values are smaller and occur at a later point in time. At 290 °C and 300 °C, the peak values are reached relatively fast, whereas the heat flow curve at 280 °C already shows a significantly more sluggish precipitation reaction. For example, between 290 °C and 280 °C, the difference in the peak time is about 500 s (roughly a factor of 5). At the temperature of 280 °C, it seems that the precipitation was not finished after 30 min, which causes uncertainties in evaluation. At the soaking temperature of 250 °C, the precipitation signal was hardly measureable with the device used in its applied configuration.

**Figure 10.**
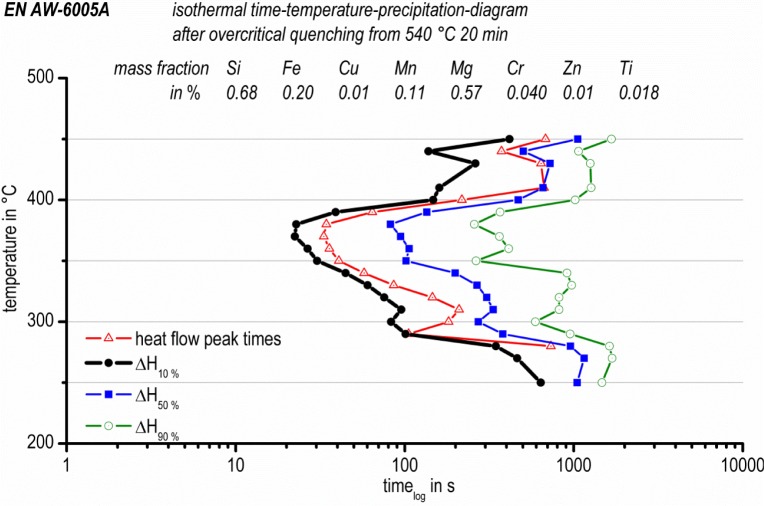
Isothermal time-temperature-precipitation-diagram of EN AW-6005A measured by *in situ* DSC experiments.

Figure 10 shows the isothermal time-temperature-precipitation diagram (TTP diagram) of the aluminum alloy EN AW-6005A, which is based on *in situ* isothermal DSC measurements. The four different datasets (peak values, 10% ∆*H*, 50% ∆*H* and 90% ∆*H*) are plotted in different colors. The four different curves have very similar shapes. In general, the whole curve shape can be described as the expected “C-curve”. However, in detail, each curve shows three local time-minima at about 440 °C, 370 °C and 300 °C, respectively. Based on the results of this study, a temperature of about 370 °C must be considered to be very critical for precipitation. At 370 °C, 10% of the precipitation reactions have already happened after about 25 s.

**Figure 11.**
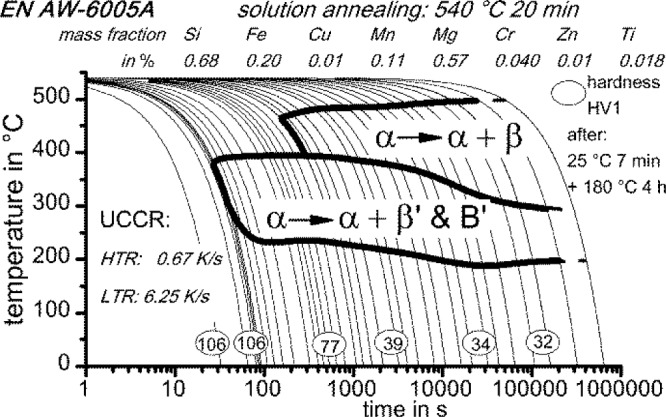
Continuous cooling precipitation diagram of the same batch of 6005A investigated in the present study [31]. HTR, high temperature; LTR, low temperature.

At 440 °C, 10% of the precipitated volume is reached after about 135 s, and at 300 °C, the same relative volume is reached after 80 s. For the high and medium temperature range, the precipitation temperatures and times can be correlated with the continuous cooling precipitation diagram in Figure 11 [31]. The same batch of EN AW-6005A and the same solution annealing parameters were applied [31]. However, during continuous cooling, only two main precipitation areas were found: within a temperature range of about 480 °C to 390 °C, the precipitation of coarse β-Mg_2_Si (up to some 10 μm in length) nucleating at large primary precipitates was detected. At temperatures between about 390 °C and 230 °C, the precipitation of precursor phases, like β’ and B’, were found. Those precipitates were much more homogenously distributed.

It might be assumed that the same types of precipitates are formed during isothermal soaking within similar temperature ranges. This is supported by the results published by Li *et al*. [14] for a similar alloy, 6063. At a isothermal temperature of 360 °C, they found rod-shaped precipitates with similar dimensions as we found for precipitation below 390 °C during continuous cooling in the alloy 6005A [31]. For a 6xxx alloy, Colley *et al.* [23] differentiate between inter- and intra-granular β-Mg_2_Si for precipitation at high or low temperatures, respectively. The 10% line for precipitation published by Colley *et al.* [23] has its critical nose at about 360 °C after 20 s, which is very similar to our results.

However, since during continuous cooling, the nucleation conditions also change continuously, it is to be expected that some differences are present compared with isothermal precipitation. For a slow cooling, first β-Mg_2_Si forms, and later on, also β’ and B’ precipitate. At that time, the amount of solutes available for the precipitation of β’ and B’ is already reduced, which is not the case for isothermal soaking at relevant temperatures. This might explain why, for isothermal soaking, three different temperature ranges for precipitation were found, contrary to the continuous cooling precipitation diagram with two precipitation areas. This finding supports similarities between crystallization and precipitation experiments, as mentioned in the introduction. Both crystallization and precipitation below a temperature range, which is critical for the related processes, are only accessible after quenching faster than the related critical cooling rate [35]. Therefore, in the present case, the investigation of the precipitation of, e.g., β” is only possible after fast quenching to an appropriate temperature and isothermal soaking.

**Figure 12.**
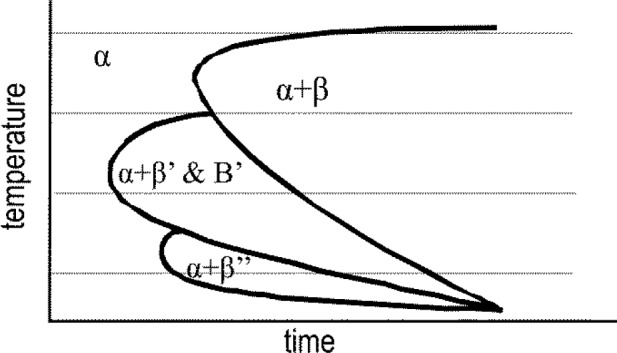
Schematic description of isothermal time-temperature-precipitation (TTP) diagram of 6xxx alloys.

According to microstructure analysis after continuous cooling [31] and the Al-Mg-Si ageing sequence [37], the precipitates at high temperatures may be β-Mg_2_Si and at medium temperatures β’ (resp. B’), and at lower temperature, precipitates like β” will form. The metastable precipitates moreover will transform with the increasing duration of isothermal soaking towards more stable phases until equilibrium is reached. Such a transformation behavior of metastable phases towards more stable ones with increasing annealing duration was also confirmed by Wang *et al.* [38], who found that “the sensitive temperature range of 6005 aluminum alloy is 280–420 °C with the nose temperature of 340 °C. Microstructure observation indicates that the supersaturated solid solution decomposes and β” precipitates during the initial stage of isothermal holding. With the prolonging of isothermal time, β’ and β form in the alloy”. Such a transformation of phases is well known and generally accepted from investigations on artificial ageing. As a consequence of these considerations, a schematic isothermal TTP diagram of 6xxx aluminum alloys based on our results in Figure 10 is illustrated in Figure 12. The above scheme of the TTP diagram is supported by Ives *et al.* [15] for the aluminum alloy 2024. In particular, they report that metastable phases after prolonged soaking transform to stable phases.

Frequently, the published isothermal TTP diagrams show a “C-curve”. This is a consequence of the interaction between diffusion and the number of nucleation sites. At high temperatures, diffusion is fast and the number of nucleation sites is low. At low temperatures, diffusion is slow and the number of nucleation sites is high. Optimal conditions for precipitation processes are located at intermediate temperatures. The three local “C-curve” noses found in the present work should therefore result from different phases or different nucleation mechanisms.

However, the heat flow curves measured here only show one peak, but no precipitation or sequence. The measuring system of the used device (Pyris 1 DSC, PerkinElmer) is suitable for relatively fast cooling rates, which is crucial to be able to perform the whole heat treatment cycle inside one device. Nevertheless, the sensitivity of this device obviously is not sufficient to resolve very small heat flows resulting from the transformation of one phase to another. Therefore, the transformation from β” → β’ → β could not be proven in the heat flow curves. For this purpose, other types of DSC, like high sensitivity CALVET-type devices, seem to be more promising. However, in order to use devices such as these for this purpose in future experiments, the possibility for fast quenching will need to be incorporated within them.

**Figure 13.**
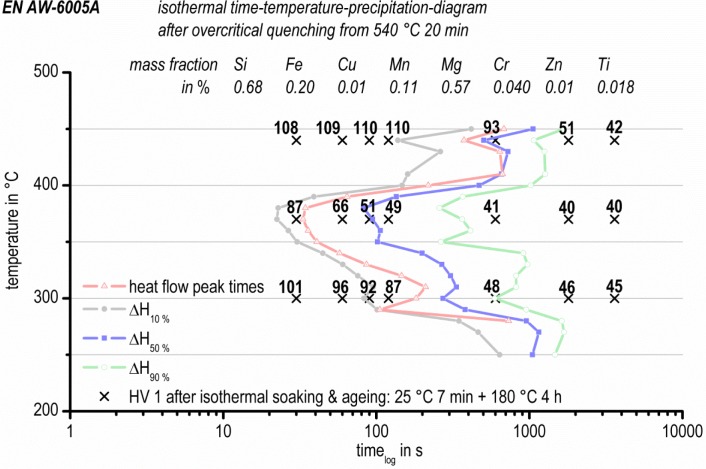
Isothermal time-temperature-precipitation diagram of EN AW-6005A, including the residual hardening potential for artificial ageing by Vickers hardness values.

In Figure 13, the isothermal TTP diagram of EN AW-6005A is plotted again, including the residual hardening potential for additional artificial ageing. Vickers hardness values for different soaking temperatures and durations (the same time-temperature profile as for DSC-measurements, plus artificial ageing; see Figure 8) are shown. The residual hardening potential qualitatively supports the DSC measurements. Hardness after ageing decreases with increasing soaking duration, due to the loss of solutes during soaking. The maximum residual hardening potential was found to be 110 ± 2 HV1, which is near the values found after overcritical continuous cooling, where the maximum residual hardening potential amounts to 106 ± 3 HV1 [31]. For high temperatures, the decrease of the residual hardening potential begins after about 120 s. At medium temperatures, the residual hardening potential decreases after 30 s of soaking. At low temperatures, the decrease of potential residual hardening starts after 60 s. After soaking for 60 min, the residual hardening potential is low, and Vickers hardness remains at a minimum level for each soaking temperature.

**Figure 14.**
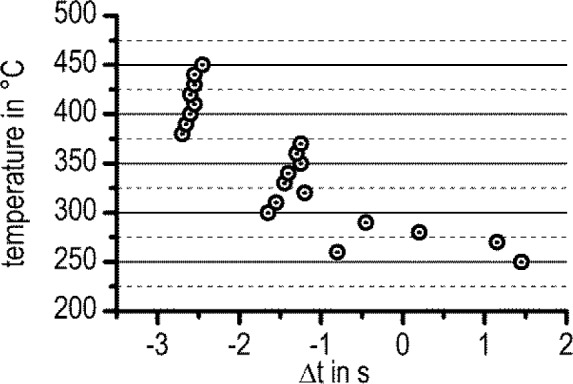
The values of Δ*t* as a function of temperature. The definition of Δ*t* is explained in Figure 4.

Finally, the reliability of the TTP diagram shall be discussed. All heat flow curves were shifted horizontally by Δ*t* (for a definition of Δ*t*, see Figure 4 and the related text). The amount of Δ*t* varied between −2.7 s to +1.4 s; see Figure 14. As the largest values of Δ*t* are present at high temperatures, where the peak times are also relatively large, the influence of the time shift, Δ*t*, on the evaluated peak times is relatively small.

**Figure 15.**
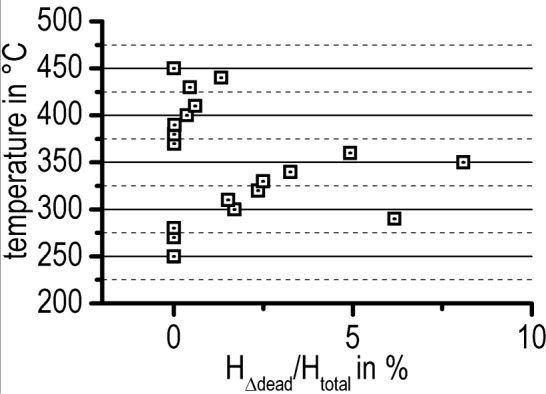
The ratio between the approximated precipitation heat, Δ*H*_dead_, and the evaluated total amount of Δ*H*_total_.

The precipitation enthalpy, Δ*H*_dead_, which was not measurable due to overshoot artefacts (Figure 4), precipitates during times *t*_dead_, which amounts to a maximum of 77 s. Compared with previous work, this dead time is relatively short. For example, the dead time in the method used by Colley *et al.* [23] amounts to five to 7 min (stated in [39], this reference is cited by Colley *et al.* [23] for the experimental details). However, it is believed that the triangle approximation of ∆*H*_dead_ overcomes this problem in a defined way. As the precipitation heat value was used for the determination of the characteristic times of the precipitation process, the ratio between the approximated contributions, Δ*H*_dead_, to the total precipitation heat, Δ*H*_total_, should be discussed. Figure 15 shows this ratio as a function of temperature. It is obvious that the ratio possesses the largest values within the temperature range most critical for precipitation. However, Δ*H*_dead_ amounts to a maximum of about 8% of the total evaluated precipitation heat. Therefore, one can conclude that the influence of the Δ*H*_dead_ approximation on the evaluation of characteristic times is relatively small.

**Figure 16.**
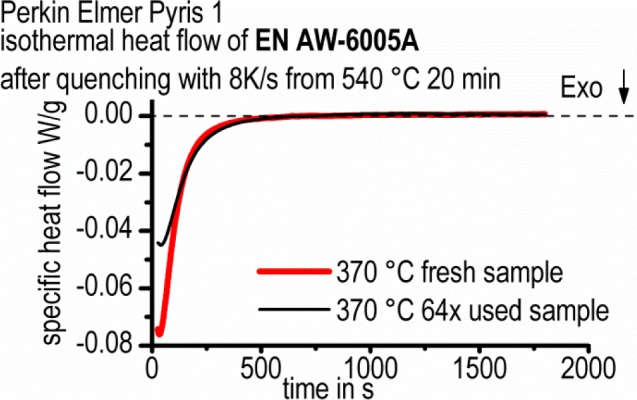
Comparison of heat flow from a fresh and a frequently measured sample.

One drawback of the present study is that all measurements were performed on only one single sample, because we were focusing on the development of a new DSC measurement method. More than 60 heat treatment cycles were applied. By a control measurement, it was found that the precipitation reaction in the heat flow curve is less intensive after 64 heat treatment cycles compared to a fresh sample (Figure 16). However, the peak time is not influenced, and besides, we are not discussing the absolute precipitation heat, but rather, precipitation heat fractions. Therefore, it is believed that the results correctly present the precipitation kinetics. Furthermore, this multiple use of only one sample also inhibits the evaluation of the precipitation volume fraction. The evaluation of the precipitation volume fraction would be preferable and is basically achievable directly from the DSC results and in detail from the precipitation heat or enthalpy under certain conditions [31,40]. However, these conditions are not fulfilled here; in particular, the total enthalpy changes due to multiple use of the sample. In the future, we will use the method developed here on a series of fresh samples.

## 4. Summary

The aim of this work was the development of a reliable method to record isothermal time-temperature-precipitation diagrams of aluminum alloys by *in situ* differential scanning calorimetry (DSC). Those diagrams may be used as a database for heat treatment simulations of the cooling step within the age hardening procedure, by applying Scheil’s additivity principle or Quench Factor Analysis, respectively [2,3]. In particular, in this work, the whole heat treatment cycle, containing solution annealing, overcritical quenching to a certain temperature and isothermal soaking, was performed in one single DSC device. Thereby, the dead time, in which precipitation might take place, but could not be recorded, was reduced to a minimum. Aluminum alloy EN AW-6005A was chosen, because its continuous cooling precipitation diagram is available for the particular batch investigated here. Thereby, the critical cooling rate, sufficient to suppress any precipitation during cooling, is known. Moreover, the here-used Perkin-Elmer Pyris 1 DSC in its applied configuration is able to cool faster than this critical cooling rate in the relevant temperature range. By this work, a data evaluation, suitable for revealing the reliable heat flow curves during isothermal soaking, is presented. Furthermore, a procedure suitable for determining the characteristic times of precipitation reactions is described. This procedure is based on peak values, but also on different fractions of the released amount of heat. Short periods of time, which were not measureable, due to device limitations, were approximated in a defined way. Additionally, the zero point of time for evaluation was defined. The time-temperature-precipitation diagram shows a general “C-curve” with three underlying weak noses. The first nose is located at 440 °C after about 135 s, the second at 370 °C after only about 22 s and the third at 300 °C after 80 s. At high temperatures, the precipitation of β-Mg_2_Si was found. At medium temperatures, the precipitation of phases, like β’ and B’, and, at low temperatures, the precipitation of β” is assumed, based on literature data. The new method enables a very detailed *in situ* analysis of precipitation during isothermal soaking. As one drawback, we admit that this procedure in its current state is only valid for medium or low concentrated aluminum alloys. As the cooling rate of the DSC device is limited at some K/s, only those medium- or low-alloyed compositions with a critical cooling rate low enough to be quenched over-critically in the Perkin Elmer Pyris 1 DSC can be studied. Nevertheless, the method developed here shows the way to go for high alloyed compositions with faster DSC devices, e.g., fast scanning chip calorimeters [30,41].

## Figures and Tables

**Table 1.** The mass fraction of alloying elements by percent, as well as the standard composition according to EN 573-3.

Mass fraction in %	Si	Fe	Cu	Mn	Mg	Cr	Zn	Ti
EN AW-6005A	0.68	0.2	0.01	0.11	0.57	0.04	0.01	0.018
EN 573-3	0.5–0.9	<0.35	<0.3	<0.5	0.4–0.7	<0.3	<0.2	<0.1
